# Cross-sectional study to assess awareness of cytomegalovirus infection among pregnant women in Germany

**DOI:** 10.1186/s12884-022-05312-8

**Published:** 2022-12-24

**Authors:** Hannah Greye, Stine Henning, Kristina Freese, Andrea Köhn, Anke Lux, Anja Radusch, Anke Redlich, Daniela Schleef, Sven Seeger, Volker Thäle, Anke Rissmann

**Affiliations:** 1 Malformation Monitoring Centre Saxony-Anhalt, Medical Faculty Otto-Von-Guericke-University Magdeburg, Leipziger Straße 44, 39120 Magdeburg, Germany; 2 Department of Obstetrics and Gynaecology, St. Marienstift Hospital Magdeburg, Harsdorfer Straße 30, 39110 Magdeburg, Germany; 3 Institute for Biometrics and Medical Informatics, Medical Faculty, Otto-Von-Guericke-University Magdeburg, Leipziger Straße 44, 39120 Magdeburg, Germany; 4 Department of Gynaecology and Obstetrics, Perinatal Centre, St. Elisabeth and St. Barbara Halle, Mauerstraße 5, 06110 Halle (Saale), Germany; 5 Department of Obstetrics and Gynaecology, Otto-Von-Guericke-University Magdeburg, Gerhart-Hauptmann-Straße 35, 39108 Magdeburg, Germany; 6 Department of Obstetrics and Gynecology, Hospital Magdeburg GmbH, Birkenallee 34, 39130 Magdeburg, Germany; 7 Department of Obstetrics and Fetal Medicine, Martin Luther University Halle-Wittenberg, Ernst-Grube-Str. 40, 06120 Halle (Saale), Germany

**Keywords:** Cytomegalovirus, Pregnant women, Knowledge, Congenital infection, Medical education, Awareness

## Abstract

**Background:**

Study aimed to assess awareness of congenital cytomegalovirus (CMV) infection and its determinants in pregnancy.

**Methods:**

Cross-sectional survey was conducted in five hospital-based maternity units in Germany. Pregnant women attending the maternity departments completed interviewer/self-administered survey questionnaire. High-risk group was defined according to contact with children under five years of age (at home or at work). Quantitative analyses using multivariable logistic regression were performed.

**Results:**

One thousand two hundred thirty-three pregnant women were included. 48.5% (*n* = 598) of women reported any knowledge about risk of CMV infection during pregnancy. CMV infection was less known than other infections or diseases (education about toxoplasmosis 95.5% (*n* = 1,177), listeriosis 60.5% (*n* = 746). 38% (*n* = 468) of participants received education about CMV. CMV awareness was associated with the level of education and employment in childcare or medical care. Only 32% (*n* = 394) of the women made use of serological screening for CMV during pregnancy (individual health service). 40.8% (*n* = 503) of pregnant women were classified as high-risk group. They had significantly higher knowledge and education about CMV, and msignificantlycant more often use of the serological screening.

**Conclusions:**

Less than half of pregnant women surveyed were aware of potential risk associated with CMV infection during pregnancy. In our study,one-third third of pregnant women made use of the serological screening for CMV.

Regarding the lack of current consensus on the role of serological CMV screening for pregnant women, hygiene preventive measures are the only evidence-based recommendation for pregnant women and knowledge increase could potentially have major public health impact.

**Supplementary Information:**

The online version contains supplementary material available at 10.1186/s12884-022-05312-8.

## Background

Cytomegalovirus (CMV), toxoplasmosis, listeriosis and chlamydia infections can negatively affect pregnancy outcomes. CMV is the most frequent cause of congenital infection. The prevalence of congenital CMV (cCMV) was determined to be 0.6 to 0.7% in industrialized countries [1, 2]. It is the leading nonhereditary cause of congenital hearing loss [2, 3]. Furthermore, cCMV can lead to substantial developmental delay in the affected child [4, 5]. The CMV seronegativity (absence of CMV IgM antibodies) is a major risk factor for cCMV [6]. In Germany, the CMV seroprevalence among women of reproductive age revealed a rate of 40 to 55% [7–11]. The intrauterine virus transmission rate in primary infection is higher than in CMV reinfection or reactivation, estimated at 30 to 50% [12–14]. Efforts are undertaken to identify possible predictors of this vertical transmission [15, 16].

The most common source of CMV infection during pregnancy is close contact with preschool-aged children at home or at work [17, 18]. They excrete the virus with urine, tears and saliva [19].

In the absence of an approved vaccine, the current consensus is primary prevention of CMV infection during pregnancy through hygiene measures to prevent infection during pregnancy [20]. Hygiene measures to prevent maternal CMV infection have been shown to significantly reduce the seroconversion rate in seronegative pregnant women [21].

Hence, knowledge about CMV is important for newly pregnant women to reduce the negative consequences of CMV infection during pregnancy [22]. Several studies have shown that most pregnant women know little about CMV infection [23]. Although the role of CMV screening during pregnancy has been discussed over the last decade [24, 25], CMV is the least known in pregnant women compared to other congenital infections or diseases [26, 27].

However, there are limited data on the awareness and knowledge of cCMV among pregnant women in Germany. The objective of this study was to investigate awareness of cCMV infection and its socio-demographic determinants in pregnant women compared to other infectious diseases during pregnancy in Saxony-Anhalt, Germany. Secondary objective was to determine the uptake of serological screening for CMV during pregnancy as an individual health service.

## Methods

Our study used a cross-sectional, non-interventional research design. The study carried out a multicentre survey using an interviewer/self-administered paper-based interview. Participants were given a choice to either complete the survey on their own, or SH assisted the participants with the survey as needed. Only one interviewer (SH) was involved in administering the survey, and thus, the process of data collection did not vary due to multiple interviewers.

The survey was conducted in five hospital-based maternity units in Saxony-Anhalt, including two university hospitals. Therefore, all pregnant women from two cities delivering at one of the five participating hospitals were eligible for the study. To be included, the woman had to be currently pregnant, be able to understand German well, and consent to participate. The hospitals 1, 2, 3 are located in Magdeburg (with approximately 2,500 live birth annually) and hospital 4, 5 in Halle (with approximately 3,500 live birth annually). Which represent 35% of annual births in Saxony-Anhalt, a central rural region of Germany.

The self-administered questionnaire included 26 questions. The first part of the questionnaire covered demographic data and pregnancy-related information, including respondents' age, ethnicity, level of education, estimated date of birth, number of pregnancies, planned or unplanned pregnancy, and folic acid use. The gestational age was dichotomized into first or second trimester versus third trimester due to the small number of women in the first trimester. In the central part, awareness of toxoplasmosis, listeriosis, chlamydiosis and CMV infection were recorded by the two questions 1) Have you ever heard of (toxoplasmosis)? 2) Have you been informed about (toxoplasmosis), and if so, who informed you? Further, it was asked about participation in the serological screening for toxoplasmosis and CMV, which is an individual health service (IGeL) in Germany. Individual health services (IGeL) are services that are not under liability of the German statutory health insurance. These serological screening have to be explicitly requested by the patient after informed consent and have to be paid out of pocket. The serological test for CMV measures IgG and IgM during pregnancy. However, in the study we did not test for seronegativity or seropositivity. Since the focus was congenital CMV, additional information was requested, including risk factors, such as working in the medical sector, working with children, and children under the age of five living in the same household. Knowledge was tested by the two questions 1) Do you think a CMV infection can be transmitted to the unborn child? 2) Which of the following are the symptoms of a newborn with congenital CMV infection? The reply options for the second question included jaundice, death, hearing disorders, mental retardation, visual disorders, and low birth weight, which are all correct. At the end, participating women were asked whether they had changed their dietary habits because of pregnancy and whether they had ever heard of syphilis, group B streptococcus, trisomy 21, spina bifida, fetal alcohol syndrome, and metabolic disorders.

The questionnaire covers socio-demographic data, pregnancy-related information, folic acid intake, known risk factors for CMV infection, general knowledge about infections during pregnancy, particularly CMV and toxoplasmosis, and attitudes toward screening for the listed infections. Detailed questionnaire in supplement.

Data were collected from July 2018 until April 2019 in all maternity departments in Halle and Magdeburg, Saxony-Anhalt, including two university hospitals. All maternity departments in the two cities were included, this should allow for a representative sample of participants from all socio-economic strata. In order to obtain a representative group of pregnant women, two different ways of recruitment were used. Each participating hospital usually offers a voluntary event once or twice a month to introduce its maternity unit to pregnant women. It was within this context that women were asked to participate in this study (population A). In addition, midwife consultations were also used in all participating hospitals, with the exception of hospital 2, to make contact with the pregnant women, defined as population B.

Descriptive statistics were computed by calculating frequencies and percentages for responses. Population A and population B were tested for significant differences in sociodemographic data and knowledge about the infections. All statistical tests were two-sided, and significance for all statistical tests was set at *p* < 0.05.

Further, based on the literature review a high-risk group of the participating women was defined. This included women with at least one of the following characteristics: children under the age of five, living in the same household; working with special need children in school, early support, children´s support, child care/welfare, hospital, emergency service. The high-risk group and average-risk group were assessed for significant differences in their knowledge about the infections and the sources of information.

We estimated the association between women’s socio-demographic characteristics and CMV awareness using odds ratios. Multivariable logistic regression was used to adjust for potential confounders identified on univariate analysis including age, ethnicity, education level, professional qualification, and employment in childcare or medical care, number of children, planned/unplanned pregnancy and trimester of pregnancy. The multivariable analysis was restricted to only those women with complete data on all variables. All women indicated knowledge and education about CMV, were defined as CMV aware (question 16 and 19 answered with yes).

Data were analyzed using the IBM SPSS Statistics (Version 26).

Ethical approval for the study was obtained from the Medical Faculty Otto-von-Guericke-University Magdeburg Ethics Committee (78/18) on 25th June 2018. The study was conducted according to good clinical practice guidelines and the principles of the Declaration of Helsinki. This research did not receive any specific grant from funding agencies in the public, commercial, or not-for-profit sectors.

## Results

### Demographic data

**Fig. 1:**
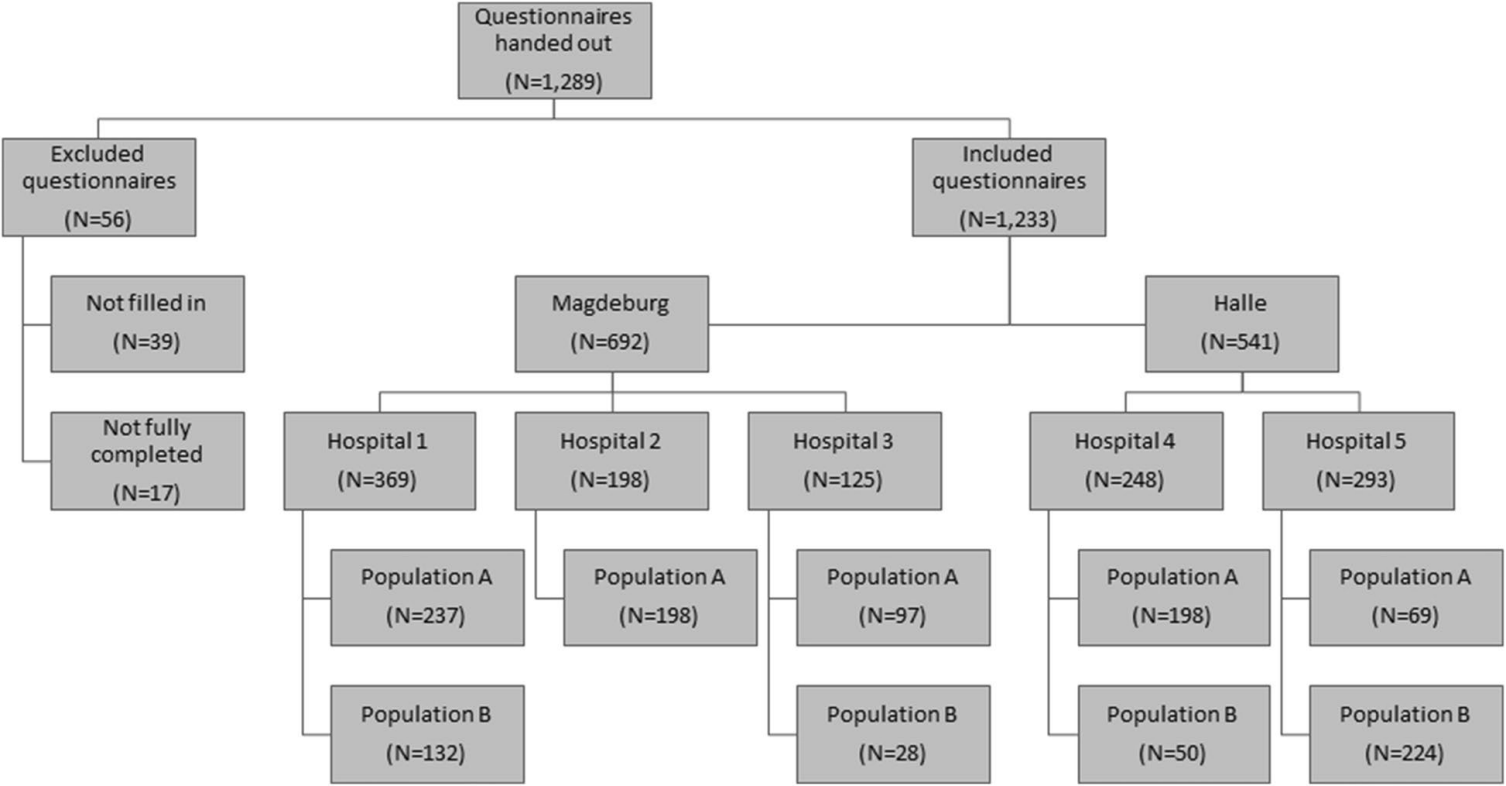
Flowchart presenting the survey participants by hospital and recruitment method

Overall, 1,289 women received the questionnaire. There were 56 questionnaires excluded from the final analysis (39 without consent and 17 not completed). A total of 1,233 women, including 799 population A and 434 population B, participated in the study. The overall response rate was 95.6%. Figure 1 shows the number of participants from each hospital.

During the study period, 41% of all pregnant women who had delivered in one of the study hospitals participated in the survey (proportion of women per hospital and more details on birth rate are given in Table 1).

**Table 1:** Overview of the proportion of women surveyed by birth rate per hospital

	Study period	Number of births^a^	Number of population A	Number of population B	Percentage (%) of women responding
Hospital 1	07/2018–03/2019	928	237	132	40%
Hospital 2	07/2018–03/2019	754	198	-	26%
Hospital 3	01/2019–03/2019	312	97	28	40%
Hospital 4	10/2018–03/2019	964	198	50	26%
Hospital 5	01/2019–04/2019	401	69	224	73%
Total		3,359	799	434	41%

^a^during time period of survey recruitment

Demographic data of the study population were similar in all five hospitals. Therefore, in the following the distinction and statistical analysis were made between the two different recruitment paths (population A and population B). Descriptive characteristics of all participants are presented in Table 2.

**Table 2:** Comparison of the demographic characteristics of study population A and B, pregnant women attending the hospital for labour planning either during presentation of the maternity ward (Population A) or at midwife consultation (Population B)

Category	Population A N (%)	Population B N (%)	*p*-value
**Total number**	799 (100)	434 (100)	
**Country of origin**			0.928
German	775 (97)	421 (97)	
European	6 (0.75)	7 (1.6)	
Non-European	15 (1.88)	5 (1.2)	
**Age group**^**a**^			0.001
17–19	12 (1.5)	9 (2.1)	
20–24	57 (7.2)	41 (9.5)	
25–29	288 (36.3)	113 (26.1)	
30–34	302 (38.1)	166 (38.3)	
35–39	116 (14.6)	85 (19.6)	
40–45	20 (2.5)	19 (4.4)	
**Highest Level of Education**			< 0.001
No graduation	2 (0.3)	7 (1.6)	
Lower secondary school	20 (2.5)	38 (8.8)	
Secondary school (O-Levels)	323 (40.4)	194 (44.7)	
Upper secondary school (A-Levels)	452 (56.6)	193 (44.5)	
**Professional qualification**			< 0.001
No qualification	15 (1.9)	30 (6.9)	
Still in education	21 (2.6)	7 (1.6)	
Up to university	412 (51.6)	249 (57.4)	
University	350 (43.8)	142 (32.7)	
**Working as healthcare professional OR in childcare**	260 (32.5)	150 (34.6)	0.438
**Trimester of pregnancy**			< 0.001
1st and 2nd	345 (43.2)	123 (28.3)	
3th	224 (28.0)	191 (44.0)	

^a^None of the participants were 16 years of age or younger

CMV knowledge, source of information, serological screening.

**Fig. 2:**
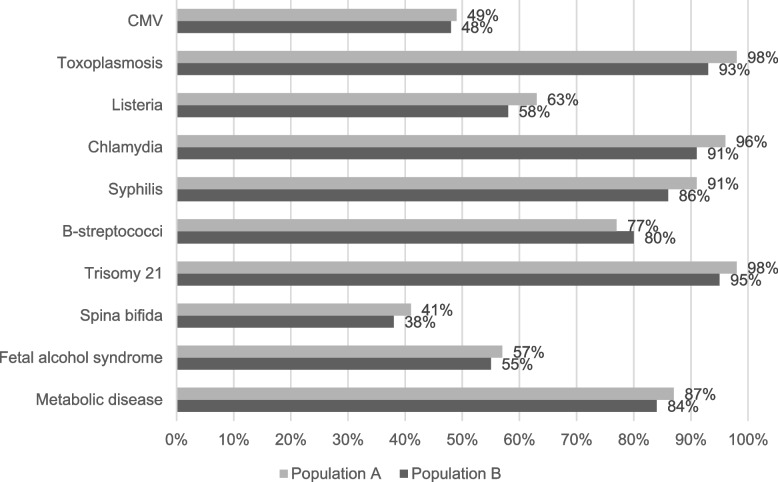
Knowledge about CMV and other congenital infections and congenital anomalies in the study population with distinction between population **A** and population **B**

Figure 2 shows the proportion of women with knowledge about CMV compared to other congenital infections and anomalies in the study population. Overall, respectively 49% (*n* = 391) and 48% (*n* = 208) of all women had any knowledge of CMV infection during pregnancy. The knowledge of CMV infection is lower than knowledge about other infections such as toxoplasmosis (98% in A, 93% in B) or listeriosis (63% in A, 58% in B). Moreover, CMV was far less known than other congenital anomalies (except spina bifida) such as trisomy 21 (98% in A, 95% in B) or fetal alcohol syndrome (57% in A, 55% in B).

Education about CMV infection was lower than about other congenital infections or malformations. Only 38% (in A *n* = 303 and B *n* = 165) of the women reported that they received any education about CMV infection during pregnancy. The proportion is lower compared to education about toxoplasmosis (94% in A, 88% in B), listeriosis (53% in A, 50% in B) and chlamydia (67% in A, 64% in B).

The sources of the women´s information included among others: attending doctors, midwife, family/friends, internet, book/journal. The women could also choose “missing value”, reported proportion was highest in CMV infection (Table 3).

**Table 3:** Sources of information according to CMV, toxoplasmosis, listeriosis, chlamydiasis in the study population

		Missing value	Treating doctor	Midwife	Family/ friends	Internet	Books/ Journals
CMV	A	60%	36%	4%	1%	8%	3%
	B	61%	36%	6%	1%	5%	4%
Toxoplasmosis	A	7%	86%	15%	15%	23%	10%
	B	12%	81%	18%	9%	17%	10%
Listeriosis	A	46%	44%	6%	5%	19%	8%
	B	50%	39%	9%	3%	15%	8%
Chlamydiasis	A	32%	61%	8%	3%	10%	5%
	B	36%	58%	9%	3%	7%	6%

**Fig. 3:**
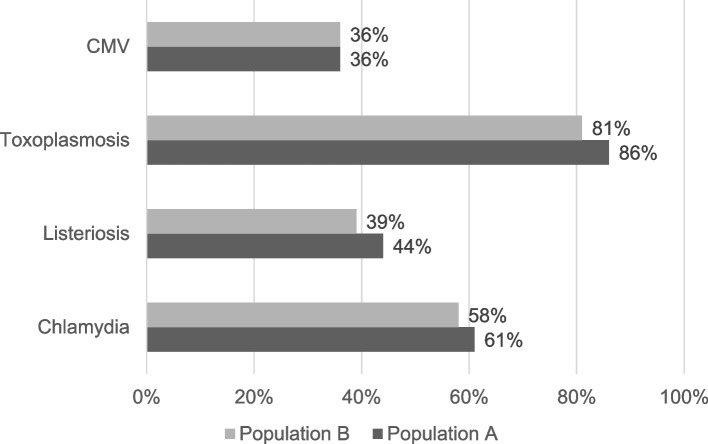
Source of information “attending doctor” reported by the study population with distinction between population **A** and population **B**

Only half of the women indicated being educated about CMV (36% in A and B) compared to toxoplasmosis (81% in A, 86% in B) (Fig. 3).

Only 33% (*n* = 264) in population A and 31% (*n* = 134) in population B made use of CMV serological screening during pregnancy. In contrast, 84% (*n* = 671 population A) and 73% (*n* = 317 population B) were tested for toxoplasmosis.

### Determinants for CMV awareness

The univariate analysis showed that maternal CMV awareness was negative associated with the level of education (OR 0.49, 95% CI 0.391–0.638), with no professional qualification (OR 0.08, 95% CI 0.025–0.272) respectively still in qualification (OR 0.5, 95% CI 0.386–0.648), and positive with employment in childcare or medical care (OR 2.46, 95% CI 1.928–3.154) as well as planned pregnancy (OR 1.66, 95% CI 1.203–2.3). The risk of being aware of CMV infection during pregnancy according to women’s socio-demographic characteristics is summarized in Table 4.

**Table 4:** Socio-demographic characteristics and maternal CMV awareness

Characteristics	Aware of CMV n (%)	Unaware of CMV n (%)	Univariate analysis		
			OR	95% CI	*P*-value
**Site**					0.704
Population A	299 (38.1%)	485 (61.9%)	1	-	
Popultion B	157 (37%)	267 (63%)	0.954	0.747–1.218	
**Country of origin**					0.6
German	446 (38.1%)	726 (61.9%)	1		
European (except German)	7 (36.8%)	12 (63.2%)	0.950	0.371–2.430	
Non-European	3 (23.1%)	10 (76.9%)	0.488	0.134–1.784	
**Age**					0.007
< 20	1 (6.3%)	15 (93.8%)	0.170	0.2–1.444	
20–29	171 (35.6%)	309 (64.4%)	1.409	0.684–2.9	
30–39	272 (41.1%)	390 (58.9%)	1.775	0.869–3.627	
≥ 40	11 (28.2%)	28 (71.8%)	1		
**Education**					< 0.001
No graduation	1 (11.1%)	8 (88.9%)	0.141	0.018–1.137	
Lower secondary school	0 (0%)	54 (100%)	0.0	0.0	
Secondary school (O-Levels)	155 (30.6%)	351 (69.4%)	0.499	0.391–0.638	
Upper secondary school (A-Levels)	298 (46.9%)	337 (53.1%)	1		
**Professional qualification**					< 0.001
No qualification	3 (7.0%)	40 (93.0%)	0.083	0.025–0.272	
Still in qualification	158 (31.1%)	350 (68.9%)	0.500	0.386–0.648	
Up to university	60 (40.5%)	88 (59.5%)	0.756	0.520–1.097	
University	231 (47.4%)	256 (52.6%)	1		
**Employment**					< 0.001
Childcare/medical care	211 (52.0%)	195 (48.0%)	2.466	1.928–3.154	
others	244 (30.5%)	556 (69.5%)	1		
**Children**					0.953
Yes	137 (37.8%)	225 (62.2%)	1.008	0.782–1.299	
No	316 (37.7%)	523 (62.3%)	1		
**Pregnancy**					0.002
Planned	373 (39.7%)	566 (60.3%)	1.664	1.203–2.3	
Unplanned	61 (28.4%)	154 (71.6%)	1		
**Trimester of pregnancy**					0.569
1./2	188 (36.9%)	322 (63.1%)	0.934	0.737–1.182	
3	267 (38.5%)	427 (61.5%)	1		

*95% CI* 95% Confidence intervall

There are no significant differences in age, country of origin and level of education comparing the high-risk group and the average-risk group of participating women in population A and B. Table 5 shows knowledge and education about the infections in the high-risk group of population A and B. The *p*-value was calculated under exclusion of questions marked with „missing value “.

**Table 5:** Knowledge, education and uptake of serological screening for various congenital infections according to risk group

	Population A (*n* = 799)			Population B (*n* = 434)		
	Average-risk group (*n* = 542)	High-risk group (*n* = 257)	*p*-value	Average-risk group (*n* = 188)	High-risk group (*n* = 246)	*p*-value
Knowledge toxoplasmosis	529 (97.6%)	253 (98.4%)	0.248	174 (92.6%)	230 (93.5%)	0.49
Education toxoplasmosis	502 (92.6%)	245 (95.3%)	0.043	164 (87.2%)	217 (88.2%)	0.986
Toxoplasmosis serological screening	462 (85.2%)	212 (82.5%)	0.338	136 (72.3%)	183 (74.4%)	0.986
Knowledge listeriosis	334 (61.6%)	171 (66.5%)	0.136	99 (52.7%)	152 (61.8%)	0.057
Education listeriosis	279 (51.5%)	147 (57.2%)	0.244	90 (47.9%)	127 (51.6%)	0.356
Knowledge CMV	228 (42.1%)	162 (63.0%)	< 0.001	68 (36.2%)	141 (57.3%)	< 0.001
Education CMV	174 (32.1%)	133 (51.8%)	< 0.001	58 (30.9%)	106 (43.1%)	0.008
CMV serological screening	148 (27.3%)	112 (43.6%)	< 0.001	47 (25.0%)	86 (35.0%)	0.034
Knowledge chlamydiasis	516 (95.2%)	252 (98.1%)	0.014	169 (89.9%)	224 (91.1%)	0.486
Education chlamydiasis	346 (63.8%)	186 (72.4%)	0.043	122 (64.9%)	154 (62.6%)	0.616

There were significant differences between the high-risk and average-risk group in terms of knowledge, education and utilisation of serological testing.

Table 6 shows the source of information about CMV in the high-risk group compared to the average-risk group. The high-risk group was significantly more often educated by doctors, journals and “other” sources than the average-risk group.

**Table 6:** Source of information about CMV infection compared by high- and average-risk group

Source of information about CMV	High-risk group n (%)	Average-risk group n (%)	Chi-squared test: asymptotic significance	Chi-squared test: exact significance
Family/friends	2 (0.8)	2 (1.1)	0.336	0.399
Midwife	24 (4.8)	32 (4.4)	0.748	0.782
Doctor	219 (43.5)	224 (30.7)	0.000	< 0.001
Pharmacist	1 (0.2)	0 (0.0)	0.228	0.408
Counselling center	0 (0.0)	1 (0.1)	0.406	1.000
Journals/guidebooks	25 (5.0)	17 (2.3)	0.012	0.016
Internet	40 (8.0)	40 (5.5)	0.083	0.099
TV/radio	0	0		
Others	18 (3.6)	10 (1.4)	0.011	0.012

## Discussion

Our multicentre observational survey regarding knowledge and education about CMV infection during pregnancy showed that– although congenital CMV infection is the most common non-hereditary cause of neurodevelopmental disability and hearing loss in infancy- the majority (60%) of pregnant women surveyed were unaware of the risk of CMV infection. These findings are also compatible with data reported from other wealthy countries such as Canada, the Netherlands and Italy [27–29]. Nevertheless, one study from France showed 60% of women had heard of CMV infection in pregnancy and there was a positive association if the hospital where they received care provided precise counselling [30]. Data from the United States observed consistent with our data that awareness of congenital CMV and its associated sequelae is very low in pregnant women and healthcare providers [31].

To our knowledge, this is the first assessment of the awareness about CMV infection compared to other congenital infections during pregnancy in such a large sample (*n* = 1,233) of pregnant women in Germany.

The strength of our study is the high response rate (95.7%) as well as the multi-centre approach. In addition, for the first time in Germany, the study observed, knowledge and education about infectious diseases in pregnancy among a large cohort of pregnant women. Furthermore, it is the first time to observe the percentage of pregnant women make use of the serological CMV (25.0%-43.6%) and toxoplasmosis (72.3%-85.2%) screening as part of individual health service (IGeL), which is not under liability of the German statutory health insurance and has to be paid out of pocket.

Consistent with other published studies our survey collected information that pregnant women were more aware of other congenital infections such as toxoplasmosis (93%-98%) and congenital anomalies such as trisomy 21 (95%-98%) or fetal alcohol syndrome (55%-57%) [29, 32].

Data from a retrospective cohort study in Saxony-Anhalt, Germany, showed that the prevalence of congenital CMV infection ranged from 0.023 to 2.2%, depending on the associated severity of sequelae [33]. A recent published meta-analysis reported similar proportions of symptomatic infants, ranging from 0.3% to 2%, following either primary or nonprimary CMV infection during pregnancy [34], demonstrating a significant public health impact [35–37]. Cannon et al. estimated that in the United States congenital CMV lead to more children with disabilities annually than trisomy 21, fetal alcohol syndrome, or spina bifida [38].

Currently, routine CMV screening is not recommended prenatally or postnatally in Germany, as in other European countries [39]. While treatment strategies during pregnancy, including hyperimmune globulins and antiviral drugs, are still under review, hygiene counselling is a current standard of care [40]. Published data showed that education and hygiene counselling of pregnant women leads to a significant reduction in the seroconversion rate [21, 22].

However, the study population could not be considered representative of all pregnant women in Germany. The survey was conducted in all five maternity hospitals in the two largest cities in the region to allow the inclusion of women from a wide range of socio-economic backgrounds. While this was limited by the required German language skills. Nevertheless, the demographic characteristics of our study population showed that the surveyed population mainly comprised women with a higher level of education. In our data, the proportion of the "highest educational level" with A-Levels (Abitur) was 56.6%—44.5% compared to the general population with 24%—32% according to the Federal Statistical Office 2021 [41]. In our study cohort, 97% of women had Germany as their country of origin; the 2021 statistical analysis showed that 87% of all women giving birth in Germany had Germany as their country of origin [42]. But education level (O-Levels) showed negative association with CMV awareness. Therefore, awareness of CMV infection during pregnancy may be lower nationally than in this survey.

Although our study had a cross-sectional design and the interpretation of associations should remain with caution, the data indicates that pregnant women had a lack of education on risk caused by CMV infection during pregnancy while doctors and health professionals play an crucial role in providing information for pregnant women [43]. In our study only 36% of all women (43.5% in the high-risk group) indicated the doctor as a source of information regarding CMV infection compared to 81%-86% for toxoplasmosis infection. In view of the importance of hygiene counselling as a currently approved method for the prevention of congenital CMV, this is a surprisingly low proportion. This may due to low awareness of CMV risk among the attending doctors/medical professionals.

However, as a consequence of the lack of awareness, only 32% of all women (average risk group 26.7%, high-risk group 39.4%) made use of the CMV serological screening. Whereas 81% of all women (average risk group 81.9%, high-risk group 78.5%) made use of serological screening for toxoplasmosis. Beaudoin et al. were able to demonstrate that the majority of pregnant women – once informed about congenital CMV infection- opt for serological CMV screening [44].

On the other hand, the comparison of the high-risk group with the average-risk group revealed that women of the high-risk group had significant more knowledge about CMV, reported more education and showed a higher acceptance of CMV serological screening. They also correctly recognised the symptoms of congenital CMV infection.

The difference in the sources of education demonstrates that the higher knowledge in our study population is due to education by doctors as well as self-education on the internet and "other" sources. About half of the women (43.5%) in the high-risk group were informed by the doctor. Considering the high risk, only 35% and 43.6% of pregnant women respectively participated in CMV serological screening.

Ongoing research focuses on strategies to educate pregnant women or those who want to become pregnant, and what could motivate women to adopt new behaviours to prevent cmv infection [45, 46]. Hygiene counseling have an impact on the rate of CMV primary infection during pregnancy as shown in the study in France [22].

Consequently, our survey results support the strategy that health care providers (doctors) need to routinely counsel pregnant women or those who want to become pregnant about CMV risk, modes of transmission and prevention of transmission. As infants and young children are the major source of maternal infection because they shed CMV in their urine and saliva at a higher rate than adults. Therefore, the risk of maternal infection is high if a pregnant woman comes into contact with the saliva or urine of an infected child through her eyes, nose or mouth [47, 48]. Current guidelines of the German medical societies recommend for seronegative pregnant women who have direct contact with infants: not to share cutlery with infants or toddlers, washing hands after changing nappies and blowing noses, no kissing on the mouth [49]. However, from the published data, it appears that women were concerned about the effort it would take to change this habits. In particular, they were concerned about not kissing their child on the mouth to show affection [45].Accompanying strategies to raise awareness of CMV among pregnant women we would recomment the implementation of special education programmes through public health initatives [26, 50].

Future studies are needed to address gaps in our understanding on the role of determinants and women's ability and motivation to adhere to prevention strategies for congenital CMV [31, 45, 46].

## Conclusions

The results of our study indicated that less than half of the pregnant women surveyed were adequately informed about the risk of CMV during pregnancy. Given that hygiene counselling is currently the only recommended strategy for primary prevention of CMV infection during pregnancy, increased education of healthcare providers, and thus pregnant women, could reduce the burden of congenital CVM infection.

In our study, only one third of pregnant women made use of the serological screening for CMV. Findings suggest education and knowledge about congenital CMV need to be increased.

Regarding the lack of current consensus on the role of serological CMV screening for pregnant women, hygiene preventive measures are the only evidence-based recommendation for pregnant women and knowledge increase could potentially have a major public health impact.

## Supplementary Information

**Additional file 1.**

## Data Availability

The data that support the findings of this study are not openly available due to the sensitive nature of the questions asked in this study and are available from the corresponding author upon reasonable request.
