# Supplementary material for: Cross-sectional study to assess awareness of cytomegalovirus infection among pregnant women in Germany
Source: BMC Pregnancy Childbirth. 2022 Dec 24;22:964. doi: 10.1186/s12884-022-05312-8 (PMC9789651; doi:10.1186/s12884-022-05312-8)
Supplement: Supplementary file 1 — Additional file 1. [file 12884_2022_5312_MOESM1_ESM.pdf]

UNIVERSITÄTSKLINIKUM MAGDEBURG A.ö.R.

OTTO VON GUERICKE UNIVERSITÄT MAGDEBURG

Universitätsklinik für Frauenheilkunde, Geburtshilfe und Reproduktionsmedizin, Gerhart-Hauptmann-Straße 35, 39108 Magdeburg

Universitätsklinik für Frauenheilkunde, Geburtshilfe und Reproduktionsmedizin

Prof. Dr. med. Dr. h. c. S.-D. Costa
Direktor

Chefsekretariat:
Frau Eckardt
Tel.: 0391 67-17310
Fax: 0391 67-17311

Universitätsfrauenklinik Magdeburg
Gerhart-Hauptmann-Str. 35
39108 Magdeburg

Telefon: +49 391 67-17301/-02
ufk-chefsekr@med.ovgu.de
www.med.uni-magdeburg.de

Dear parents-to-be,

The Malformation Monitoring Centre Saxony-Anhalt is conducting an anonymous survey on the level of knowledge and education regarding infectious diseases during pregnancy.

The aim of this work is to analyse the knowledge and influencing factors of infectious diseases during pregnancy in order to be able to better advise and care for pregnant women in the future.

We support this project and would be pleased if you would agree to participate in this survey.

To do so, we will forward you this questionnaire, which you can easily fill out and hand in here or send back to us with a self-addressed and stamped envelope.

If you have any questions, please do not hesitate to contact us:

Fehlbildungsmonitoring Sachsen-Anhalt
Otto-von-Guericke-Universität Magdeburg
Frau Dr. med. A. Rißmann
Leipziger Str. 44, Haus 39
39120 Magdeburg

www.angeborene-fehlbildungen.com
Medizinstudentin H. Rütten, S. Henning
E-Mail: monz@med.ovgu.de
Tel.: 0391/ 67 14174
Fax: 0391/ 67 14176

Participation in the survey is voluntary. You will not be disadvantaged if you do not complete the questionnaire. The survey is anonymous, no personal data will be collected. You are free not to answer individual questions or to choose the option "no answer". The data will be collected exclusively for scientific evaluation and will not be used for other purposes. It goes without saying that the data protection regulations will be observed.

We would like to thank you very much for your cooperation!

With kind regards,

Dr. med. A. Redlich
University Women's Hospital

Dr. med. A. Rißmann
Head of Malformation Monitoring

mail: monz@med.ovgu.de web: www.angeborene-fehlbildungen.com

Survey on infectious diseases during pregnancy

1. When were you born? (MM/YYYY) ____________________________

2. What is your nationality? ____________________________

3. What school-leaving qualification do you have?
   □ None □ Secondary school □ Realschule □ Grammar school

4. What professional qualification do you have? (Please mark with a cross where applicable)
   □ Apprenticeship (training)
   □ Vocational college/trade school or similar.
   □ Technical college (master school/technical academy)
   □ University/University of Applied Sciences
   □ Other educational qualification
   □ Still in training (apprentice/student)
   □ No vocational qualification

5. Do they work in a childcare or healthcare setting?
   □ Yes □ No
   If so, where?
   □ Day nursery □ Primary school □ Health care centre □ Clinic/outpatient
   □ Surgery/Practice □ other ____

6. Do you work with animals or have contact with pets?
   □ Yes □ No

7. When is the calculated date of birth? ____/ ____/ _______
   Or in which week of pregnancy (GA) are you currently? __________weeks GA

8. From which week of pregnancy were you aware of the pregnancy? ___________weeks GA

9. Was your pregnancy... □ planned ? □ unplanned ? □ not specified

10. Did you take folic acid or vitamin supplements before conception?
    □ Yes □ No □ No indication
    If yes, how long before? _ _ weeks

    Did you take folic acid or vitamin supplements during pregnancy?
    □ Yes □ No □ No indication
    If yes, in which week of pregnancy did you start taking it? ________weeks GA

11. Have you ever heard of "toxoplasmosis" before today?
    □ Yes □ No □ No information/I do not know

12. Have you used the IGeL services (self-pay laboratory test) for testing the toxoplasmosis status?

□ Yes □ No □ No indication

13. Have you been informed about the risks of toxoplasmosis infection during pregnancy?

□ Yes □ No □ No indication

If yes, who informed you? (Multiple answers possible)

□ Family/friends □ Midwife □ Doctor □ Pharmacist □ Counselling centres Newspapers/books □ Internet Radio/TV

□ other source of information: ______________________________

14. Have you ever heard of "listeriosis" before today?

□ Yes □ No □ No indication

15. Have you been informed about the risks of listeria infection during pregnancy?

□ Yes □ No □ No indication

If yes, who gave you the information? (Multiple answers possible)

□ Family/friends □ Midwife □ Doctor □ Pharmacist □ Counselling centres Newspapers/books □ Internet Radio/TV

□ andere Informationsquelle:______________________________

16. Have you ever heard of "cytomegalovirus" (CMV) before today?

□ Yes □ No □ No indication

17. Have you used the IGeL service (self-pay determination at the attending doctor) to test the CMV status?

□ Yes □ No □ No indication

18. Do you know if you have protective antibodies against CMV infection?

□ Yes □ No □ No indication

19. Have you been informed about the risks of CMV infection during pregnancy?

□ Yes □ No □ No indication

If yes, who informed you? (Multiple answers possible)

□ Family/friends □ Midwife □ Doctor □ Pharmacist □ Counselling centres Newspapers/books □ Internet Radio/TV

□ other source of information: ______________________________

mail: monz@med.ovgu.de web: www.angeborene-fehlbildungen.com

20. To your knowledge, can a CMV infection be transmitted from the mother to the unborn child?

□ Yes □ No □ No information/I do not know

21. Please tick below what you think the child may show for symptoms of congenital CMV infection (multiple answers possible)

□ Jaundice □ Death □ Hearing disorders □ Mental retardation
□ Visual disturbances □ low birth weight

22. Have you ever heard of "chlamydia" before today?

□ Yes □ No □ No information/I do not know

23. Have you been informed about the risks of chlamydia infection during pregnancy?

□ Yes □ No □ No indication

If yes, who informed you? (Multiple answers possible)

□ Family/friends □ Midwife □ Doctor □ Pharmacist □ Counselling centres
Newspapers/books □ Internet □ Radio/TV □ Other source of information:

____

24. Have you changed your eating habits during pregnancy?

□ Yes □ No □ No indication

If yes, what have you changed? (please note)

____

____

____

____

____

Please tick below which diseases you have already heard about:

□ Syphilis

□ B-streptococcus

□ Down's syndrome

□ Spina bifida

□ Fetal alcohol syndrome

□ Metabolic diseases

And now a few questions about previous pregnancies ...

26. Do you already have children?

□ Yes □ No □ No answer How many? _____

If yes, how many children between the ages of 0 and 5 live in your household?

□ 0 □ 1 □ 2 □ 3 □ 4 □ 5

Further comments/messages: ____

____

____

____

____

____

____

____

Thank you for taking part in the survey!

Malformation Monitoring Centre Saxony-Anhalt
Medical Faculty, Otto-von-Guericke-University Magdeburg
Leipziger Str. 44/ House 39
39120 Magdeburg

Tel: +49-391-6714174 fax: +49-391-6714176
Mail: monz@med.ovgu.de web: www.angeborene-fehlbildungen.com
